# Neurological Aspects of HIV-1/HTLV-1 and HIV-1/HTLV-2 Coinfection

**DOI:** 10.3390/pathogens9040250

**Published:** 2020-03-28

**Authors:** Abelardo Q.-C. Araujo

**Affiliations:** 1 Evandro Chagas National Institute of Infectious Diseases (INI-FIOCRUZ), Brazilian Ministry of Health, Rio de Janeiro 21040-360, Brazil; abelardo@ufrj.br; 2 Deolindo Couto Institute of Neurology (INDC), the Federal University of Rio de Janeiro (UFRJ), Rio de Janeiro 22290-140, Brazil

**Keywords:** HIV, HTLV, AIDS, HAM/TSP, immunodeficiency, myelopathy, progression, T lymphocytes, transmission, coinfection

## Abstract

Simultaneous infection by human immunodeficiency viruses (HIV) and human T-lymphotropic viruses (HTLV) are not uncommon since they have similar means of transmission and are simultaneously endemic in many populations. Besides causing severe immune dysfunction, these viruses are neuropathogenic and can cause neurological diseases through direct and indirect mechanisms. Many pieces of evidence at present show that coinfection may alter the natural history of general and, more specifically, neurological disorders through different mechanisms. In this review, we summarize the current evidence on the influence of coinfection on the progression and outcome of neurological complications of HTLV-1/2 and HIV-1.

## 1. Introduction

The human immunodeficiency viruses (HIV-1 and HIV-2) are lentiviruses that are highly replicative and cytopathic against cells of the immune system. HIV-2 is not as pathogenic as HIV-1 and has a more restricted geographical distribution [1]. They are transmitted mainly through unprotected (condomless) sexual intercourse and from mother to child [2]. Although horizontal transmission through contaminated blood products can still be found, donor screening has made this route less common. Infection among people who inject drugs (PWID) and share used needles and paraphernalia is still frequent. HIV infection has a pleomorphic clinical profile, affecting almost every part of the body. The prevalence of opportunistic infections and clinical manifestations of HIV-AIDS has waned significantly since the introduction of combined antiretroviral therapy (ART), mainly in countries with easy access to testing and treatment. This is far from being universal since southeast Asia and southern Africa remain hotspots for new HIV infection, which goes undetected until AIDS has developed [3]. 

HIV-1 causes severe immunodeficiency in humans. Before the advent of ART, HIV-infected patients suffered from high morbidity and mortality. Although this is no longer the case, at-risk groups remain [3]. 

The human T-cell lymphotropic viruses type 1 (HTLV-1) and type 2 (HTLV-2) are structurally similar retroviruses belonging to the genus deltaretrovirus [1]. Unlike HIV-1, which is highly cytopathic, HTLV1/2 cause proliferation of the infected cells, mainly T lymphocytes [4]. HTLV-1 has a unique tropism for CD4+ cells, while HTLV-2 favours CD8+ T lymphocytes [5]. These viruses are mostly transmissible by breast-feeding and by unprotected sexual intercourse [6]. Transplacental and intrapartum transmission are extremely rare [7].

HTLV-1/2 can also be acquired through contaminated blood, through transfusion, or by sharing paraphernalia for injecting drugs. The HTLV transmission efficiency is lower than that of HIV [1]. The lifetime risk of developing disease after HTLV-1 infection is considered low (less than 5%), but strong longitudinal data on this assumption are still lacking [8]. The identification of asymptomatic infected individuals is of public health importance because they are active transmitters of the infection. 

HTLV-1 is endemic in some restricted regions of Japan, South America, sub-Saharan Africa, the Caribbean, Iran, Romania and Melanesia. More recently, central Australia has been detected as the region with one of the highest recorded prevalence of HTLV-1 worldwide, with the infection of more than 40% of adults [1,9]. 

HTLV-1 causes lymphoproliferative malignancies and many immune-mediated disorders. Adult T-cell leukaemia/lymphoma (ATLL) is the typical malignancy associated with HTLV-1. ATLL is a clinically pleomorphic malignancy of mature T-lymphocytes [10]. 

Although more accurate data are lacking, it is believed that 5 to 10 million people may be carriers of HTLV-1 worldwide [11]. Throughout the world HTLV-2 infection is more prevalent among PWID, being particularly more common than HTLV-1 in people living with HIV-1 who also inject drugs [1]. HTLV-2 can also be found with a high prevalence in certain isolated Amerindian groups [12].

It is not yet clear how pathogenically important HTLV-2 is for humans, although some faint evidence points to its possible role in some lymphoproliferative and neurological disorders [12]. 

Because HIV-1 and HTLV-1 and 2 spread similarly, the simultaneous infection by more than one virus should not be uncommon, particularly in areas where both are concurrently endemic. PWID are at higher risk of infection with blood-borne viruses and even coinfection. This occurs not only in HIV-1/HTLV-1/2 but also in HIV-1/HCV coinfection [13].

The prevalence of coinfection by HIV-1 and HTLV-1 varies widely, depending on the group of individuals and the region of the study. It can be as high as 10.9%, in a prospective screening of 2678 individuals from an HIV-1 cohort from Rio de Janeiro, Brazil [14], or as low as 0.11% in a prospective screening of 6331 pregnant women from French Guyana [15]. The prevalence of HIV-1/HTLV-2 coinfection also varies according to the group studied and the region of origin of these individuals. It ranges from 0.005% in a prospective screening of 20,518 pregnant women from different hospitals from Spain [16] up to 29.9% in a cross-sectional study of PWID screened for HIV-1 from Buenos Aires, Argentina [17].

Studying the possible interactions between these viruses, at the cellular and molecular level, as well as the clinical and prognostic implications of this phenomenon, becomes of paramount scientific and practical importance. Although this review focuses on the neurological outcomes of people living with HIV-1/HTLV-1 and HIV-1/HTLV-2, general aspects of coinfection are also briefly addressed.

## 2. Neurological Outcomes of HTLV-1, HTLV-2 and HIV-1 Single Infections

HIV-1 and HTLV-1 cause a variety of neurological manifestations. HIV-1 causes neurological disease through direct or indirect mechanisms; the latter by inducing an immunodeficiency state that leads to the emergence of opportunistic infections (OIs). HTLV-1, in turn, causes a group of immune-mediated diseases known as a whole as the “HTLV-1 neurological complex” [18]. Among those manifestations, the more common is the HTLV-1-associated myelopathy/tropical spastic paraparesis (HAM/TSP) [8]. 

The most common neurological diseases caused by HIV are opportunistic infections (OIs), neurocognitive disorders (HAND), peripheral neuropathy (PN), vacuolar myelopathy, and CD8+ T-cell encephalitis [19]. Although less frequent, myositis and primary central nervous system (CNS) lymphoma are important causes of disability and should always be remembered in the differential diagnosis of HIV-infected patients with neurological complaints.

The introduction of ART in the treatment of HIV-1 infection has led to a significant decrease in HIV-1 morbidity and mortality. At the same time, several new inflammatory syndromes (so-called immune reconstitution inflammatory syndromes—IRIS [19]) have also been described. IRIS usually occurs after reversal or improvement of immunosuppression after initiating ART. IRIS can affect any organ or system, and neurological manifestations appear in 0.9–1.5% of infected individuals after ART. A detailed description of IRIS and its neurological consequences is beyond the scope of this Review [19].

Although the overall prevalence of HIV has steadily increased in recent years, the incidence of OIs in infected people has decreased since the advent of ART. Neurotoxoplasmosis, cytomegalovirus encephalitis, neurotuberculosis, cryptococcal meningitis, and progressive multifocal leukoencephalopathy are common CNS OIs associated with AIDS. Less common causes of OIs like varicella zoster, herpes simplex, and neurosyphilis should also be considered in the differential diagnosis of HIV infected individuals.

The risk of OIs will be greater, the higher the degree of immunosuppression of patients [13]. CD8+ T-cell encephalitis is another neurological disease associated with HIV-1. It is a severe and progressive leukoencephalitis characterized by rapid cognitive decline, headaches and seizures. Cerebrospinal fluid (CSF) corroborates the diagnosis by revealing an increase in proteins associated with a pleocytosis of CD8+ lymphocytes [20].

Since the introduction of ART, PN has become the most common neurological complication in HIV-positive patients. That is due to the significant increase in the survival of these individuals. PN affects 30% to 67% of infected persons. Generally, the disease has a subclinical evolution, with subtle changes in the neurological examination. The most common subtypes of PN are distal symmetrical polyneuropathy (HIV-DSP) and the antiretroviral toxic neuropathy. HIV-DSP is characterized by distal paraesthesias and numbness, which can be painful or not, affecting hands and feet symmetrically. Generally, HIV-DSP starts in the lower limbs being typically associated with the loss of deep tendon reflexes. Nerve biopsies will show the presence of inflammatory infiltrates [19].

HIV-associated dementia used to be more frequent before the advent of ART, affecting 20–30% of infected individuals. Although ART has drastically reduced the frequency of dementia to less than 5%, the occurrence of other neurocognitive manifestations in general (i.e., HAND) remains significantly high. The severe dementia observed in the pre-ART era has been replaced by mild to moderate neurocognitive disorders. HAND usually manifests as a combination of motor dysfunction, slow reaction times and impaired verbal fluency. Subtler cognitive complaints, such as prospective memory loss, which persist even after initiation of ART is still a problem that can impact medication adherence and everyday functioning. Recent data have shown that the prevalence of HAND as a whole, especially with mild cognitive deficits, can affect up to 50% of infected individuals [19].

The spinal cord used to be commonly involved in the HIV-infected population before the ART era. Myelopathy used to be frequent in the past, developing in one-third of patients [21]. Nowadays, this complication became less frequent. The classical myelopathy associated with HIV-1 is the so-called vacuolar myelopathy, which is a chronic progressive disease, typically associated with advanced immunosuppression [22]. An acute form of myelitis, with motor, sensory and autonomic deficits below the level of the lesion can be rarely found in infected individuals at the time of seroconversion. It can occur as the first manifestation of the infection in a still immunocompetent host. It resembles classical transverse myelitis [23].

The main neurological complication of HTLV-1, HAM/TSP, is most frequent in women in their third and fourth decades of life. The onset is usually subacute and insidious with weakness and stiffness of the legs, accompanied by urgency and increased frequency of urination. The neurological examination shows spastic paraparesis, hyperreflexia, and Babinski sign. Except for the involvement of deep sensory loss, mainly in more advanced cases, the superficial sensory testing is usually normal, although patients may complain of subjective paresthesiae. Higher HTLV-1 proviral loads are typical in the disease when compared to asymptomatic carriers [24]. Higher proviral loads correlate with stronger and specific anti-HTLV-1 cytotoxic T-cell responses. That exacerbated immune response is determinant for the etiopathogenesis of HAM/TSP [8]. Pathologically, the spinal cord, particularly the posterior columns and corticospinal tracts of the thoracic level, is the main affected region in HAM/TSP. Perivascular lymphocytic infiltration of the cord is seen, particularly in the early phases of the disease. After a few years, this inflammatory infiltrate is progressively replaced by gliosis. The release of cytokines and lymphokines by activated cytotoxic T cells is critical for the development of HAM/TSP. There is chronic and permanent damage to the surrounding glial, and neuronal tissue in a mechanism referred to as bystander damage [25]. Similar to ATLL, HAM / TSP is the consequence of a highly selective process that occurs in only a fraction of infected individuals. Genetic factors determine the individual predisposition to develop specific HTLV-related diseases. Therefore, some HLA alleles have been identified as protective, while others as contributory to the development of the disease [26,27].

Although there are some claims that HTLV-2 per se causes neurological disease, such as myelopathy or cerebellar ataxia, this has never been proven. Most reports come from single case reports or small case series with many confounders such as coinfection with HIV-1 and/or active PWID [28]. 

## 3. Virological Results of HIV-1/HTLV-1 and HIV-1/ HTLV-2 Coinfection

There has been much evidence showing potential direct and indirect interactions between HIV-1 and HTLV-1 and 2:

- HTLV-1 virions and proteins upregulate HIV-1 infection by activating CD4+ T cells [29,30,31,32];
- In cultures of peripheral blood mononuclear cell (PBMC), HIV-1 and HTLV-1 can activate each other [33]. There is an increase in antigen production and elevation of concentrations of HTLV-1 mRNA expression following coinfection with HIV-1 [34];
- Coinfection of T-cell tropic HIV-1 and HTLV-1 cultured macrophages increases HIV-1 replication, while coinfection with HIV-1 macrophage-tropic strains upregulates HTLV-1 [35];
- The Tax protein of HTLV-1 stimulates HIV-1 replication through activation of HIV-1 LTR [36].

Several pieces of evidence suggest that HTLV suppress the replication of HIV-1 by releasing CC chemokines [30,37,38,39]. This mechanism may stimulate the innate immunity against HIV-1 through the modification of CCR5/HIV-1 binding and, consequently, hindering the progression of HIV-1-related disease in coinfected individuals [40]. Some soluble factors released by HTLV-1 may increase or suppress HIV-1 infection. In coinfected individuals on ART, an increase in the prevalence of HTLV-1-associated neurological disease can be observed as ART can control the disease by HIV-1 but has very little influence on HTLV-1 expression. 

The role of HTLV-1/HIV-1 coinfection in accelerating AIDS is still controversial. A significant problem when analyzing the literature is that it is relatively scarce with significant observations mostly derived from the pre-ART era. There are suggestions that there may be an induction to HIV-1 replication in the presence of HTLV-1, and this may accelerate the evolution towards AIDS [41,42,43]. These findings have not been confirmed by others, however [44,45,46]. The existence of different HIV-1 phenotypes could be one of the explanations for such disparities [47]. Contrary to what appears to occur with HTLV-1 coinfection, HTLV-2 seems to play a protective role in the development of AIDS. Coinfection with HTLV-2 is associated with an increase in survival and to a later progression to AIDS [48,49]. That is probably due to the maintenance of normal CD4+ and CD8+ lymphocyte counts, reduced replication of HIV-1 and increased immunological activation. The percentage of long-term AIDS nonprogressors in HTLV-2/HIV-1 coinfection is significantly superior to what is found in HIV-1 single infection [42]. This beneficial effect can be explained, at least in part, by the HTLV-2 induction of chemokine and cytokine production, contributing to a predominance of the protective Th1 response in contrast to a more deleterious Th2 response [42,50,51].

When comparing HTLV-1 proviral loads among groups of HAM/TSP patients, HIV-1/HTLV-1 coinfected individuals, and HTLV-1 asymptomatic carriers, proviral loads tend to be higher in those with HAM/TSP. There is no significant difference in proviral loads or CD4+ lymphocyte counts between monoinfected and coinfected individuals. Knowing that neurological and inflammatory diseases are typically associated with higher proviral loads, these results imply that coinfection should not induce or even accelerate the development of these diseases [52].

## 4. Clinical Aspects of Coinfection

The impact of coinfection with HTLV-1 and 2 on the progression of AIDS is still controversial [1,53]. There are several reasons for these discrepancies: a small number of patients studied, inaccuracy in determining the duration of infection by each virus, and, in many cases, lack of discriminative diagnosis between HTLV-1 and HTLV-2. A multicenter report [54] showed that HIV-1/HTLV-2 coinfection did not influence the progression to AIDS or favour higher mortality. Another study in PWID coinfected with HIV-1/HTLV-2 was not able to identify higher morbidity and mortality, suggesting that HTLV-2 is not a clinically pathogenic agent within the context of coinfection [55]. Another study [56], however, showed that individuals coinfected with either HTLV-1 or HTLV-2 tended to develop AIDS more frequently than those HIV-1 infected controls without coinfection. These conflicting results are probably due to methodological differences. The first two studies specifically selected HTLV-2 coinfected individuals, while the second one was chiefly based on a mixed HTLV-1 or 2 infected populations but with a preponderance of HTLV-1.

Several studies have shown that coinfection accelerates the progression to AIDS and worsens the prognosis of OIs [41,42,43]. It is not yet clear whether HIV-1/HTLV-1 coinfection predisposes to ATLL [57] or interferes with its prognosis [24]. There are only isolated reports of ATLL development in coinfected individuals [58]. Many studies have suggested that HTLV-2 coinfection in PWID have a protective effect against the progression to AIDS [59] and, as already mentioned, it may be associated with a “long-term nonprogressive” phenotype. In summary, HTLV-2 appears to differ from HTLV-1 on its consequences and pathogenicity in the HIV-1 coinfected host, having more benign consequences. 

Individuals coinfected with HTLV-1 tend to be in more advanced stages of HIV-1 infection when compared to those living with HIV-1 alone [60]. Coinfected patients had higher levels of CD4+ cells, corroborating the well-known fact that HTLV-1 stimulates the proliferation of CD4+ lymphocytes. Other studies in patients coinfected with HIV-1/HTLV-1 show a higher CD4+ and CD8+ count when compared to mono-infected individuals [61]. HIV-1/HTLV-1 coinfection appears to be significantly correlated with shorter survival, despite adequate levels of CD4+ cells [62]. Therefore, monitoring the ART response using the CD4+ T cell count as a parameter may not be a useful approach in the presence of coinfection [63]. HTLV-1 infection results in increased T lymphocyte proliferation, which is usually balanced by a higher rate of infected cell death due to immune surveillance. Coinfection with HIV-1/HTLV-1 leads to overproduction of poorly functioning lymphocytes, providing an environment for high levels of HIV-1 virion production [57].

The utility of antiretrovirals active against HIV-1 to treat infection by HTLV-1/2 infection has been highly disappointing. Although some of them may have shown some effectiveness in vitro, their clinical usefulness has not been confirmed in infected individuals [64,65,66,67,68]. Their clinical efficacy in coinfected has not been formally tested so far. 

## 5. Neurological Consequences of Coinfection

HIV-1 and HTLV-1 and 2 are well-known neuropathogens [69,70]. Since 1991, it is known that there is an increased prevalence of a HAM/TSP-like myelopathy in patients coinfected with HIV-1/HTLV-1 [71]. Beilke et al. validated this finding when they described a 9.7% frequency of HAM/TSP among HIV/HTLV-1 coinfected individuals [72]. It is not yet clear whether simultaneous HIV-1 infection may predispose to the development of neurological diseases associated with HTLV-1 or whether HTLV-1 increases the chances of developing HIV-1 neurological complications [1,71]. Table 1 summarizes the main neurological outcomes of HIV-1/HTLV-1 and HIV-1/HTLV-2 coinfection.

Some authors have observed a significantly higher proportion of HIV-1/HTLV-1 coinfected individuals who developed myelopathy and peripheral neuropathy when compared with patients infected by HIV-1 alone [73]. In these cases, it was not possible to establish the role of each virus in the etiopathogenesis of each disease. Similarly, it was not possible to be sure that all patients who developed myelopathy had HAM/TSP since not all of them met the diagnostic criteria for this disease [49]. A more recent study found a prevalence of 10.9% of HIV/HTLV coinfection in a cohort of HIV-positive individuals from an HTLV endemic area. There was a higher risk of neurological diseases in these individuals. Also, the use of ART neither protected its users from the development of neurological diseases nor influenced the proviral HTLV load or the patients’ CD4+ lymphocyte levels [14].

The risk of HTLV-1 infected individuals developing HAM/TSP over their lifetime is less than 5% [8]. These rates appear to be higher in coinfected individuals and this increased risk may be due to increased activation of HTLV-1 infected lymphocytes [34]. It is well known that HTLV-1 proviral loads may increase during post-ART immune reconstitution. This fact is probably due to an expanded pool of HTLV-1 infected CD4+ cells [74]. It is still unclear whether ART has any influence in the pathogenesis of HTLV neurological disease. The prolonged life expectancy induced by ART may lead to an increase in the prevalence of neurological complications, such as myelopathy or peripheral neuropathy.

Neurological manifestations other than HAM/TSP may be associated with HTLV-1 [75]. These include polymyositis, polyneuropathy, motor neuron disease, cognitive impairment, neurogenic bladder and dysautonomia. The development of these disorders is also associated with higher proviral loads [76]. Whether HIV-1/HTLV-1 coinfection alters the natural history or the tendency to develop one of these manifestations of the HTLV-1 neurological complex is still uncertain [18].

In addition to the chronic progressive HAM/TSP, HIV-1/HTLV-1 coinfection may also be linked with acute transverse myelitis. That has recently been described in a coinfected and yet immunocompetent patient. Therefore, coinfection should be searched whenever someone is faced with a case of acute transverse myelitis [77].

Infection with HTLV-2, although less frequent when compared with HTLV-1, can also be associated with neurological disorders. Berger et al. and Rosenblatt et al. were the first to describe HAM/TSP-like disease in patients coinfected with HIV-1/HTLV-2 [71,78]. HIV-1/HTLV-2 coinfection may also be associated with a higher prevalence of peripheral neuropathy, particularly with cases of predominantly sensory polyneuropathy (PSP) [79]. These patients with PSP show proviral loads significantly higher than those seen in coinfected patients but without polyneuropathy [29,79]. A cohort study of HIV-HTLV-1 coinfection from Brazil found similar results: polyneuropathy was more common in coinfected individuals than in singly infected patients [14] (Table 1).

## 6. Conclusions

Human retroviruses share common routes of transmission; therefore, coinfection is not unexpected. The advent of ART significantly reduced HIV-1 morbidity and mortality, making patients live better and longer. However, this increase in survival may result in a higher prevalence of neurological manifestations among coinfected patients. 

Higher rates of neurological disease are found in coinfected individuals. The most remarkable neurological effects of HIV-1/HTLV-1 and HIV-1/HTLV-2 coinfection are myelopathy (related to HIV-1/HTLV-1 coinfection) and PN (in association with either HIV-1/HTLV-1 or HIV-1/HTLV-2 coinfection). Current antiretroviral therapies have shown no proven effect either in HTLV-1/2 singly infected individuals or in coinfected patients.

Finally, there is a scarcity of recent clinical and neurological studies in coinfected patients, with most studies describing either small samples or having a short follow-up period. Only long-term prospective studies with large numbers of patients may confirm the real prevalence or even reveal new neurological complications in these individuals. In the meantime, physicians involved in the follow-up of these patients should remain alert to the appearance of old or new clinical manifestations associated with the simultaneous infection by HIV-1 and HTLV-1/2.

## Figures and Tables

**Table 1:** Neurological aspects of HIV-1/HTLV-1/2 coinfection (adapted from [68]).

Type of Study	Population	Outcome	Reference
**Cross-sectional case-control of HIV-1 patients on ART**	47 HIV-1/HTLV-1/2 coinfected (34 HTLV-1, 3 HTLV-2, 5 HTLV-1 and 2, 5 indeterminate) vs. 381 HIV-1 monoinfected randomly selected	12/34 (35.2%) HIV-1/HTLV-1 had myelopathy (*p* < 0.01) 12/47 (25%) had PN (*p* = 0.01) 2/381 (0,5%) HIV-1 had myelopathy 52/381 (13%) had PN	Silva et al., 2012
**Prospective longitudinal observational**	38 HIV-1/HTLV-1 over 9 years	6/38 (15.7%) developed myelopathy	Casseb et al., 2008
**Prospective longitudinal observational**	41 HIV-1/HTLV-1 over 7 years	4/41 (9.7%) developed myelopathy	Beilke, 2005
**Prospective cross-sectional case-control**	31 HIV-1/HTLV-1 vs. 118 HIV-1	11/15 (73%) had myelopathy (OR 14.3, *p* = 0.0004) 10/62 (16%) had myelopathy	Harrisson, 1997
**Cross-sectional case-control**	39 HIV-1 with PN on EMG 266 HIV-1 without PN	12/39 (30.8%) coinfected by HTLV-2 (*p* = 0.001) 22/266 (8.3%) coinfected by HTLV-2	Zehender, 1995
**Prospective case-control**	30 HIV-1/HTLV-1 with a median follow-up of 28.5 months vs. 60 HIV-1	12/30 (40%) developed PN (*p* = 0.004) 8/60 (13.3%) developed PN	Zehender, 2002

HIV, human immunodeficiency virus; HTLV, human T-lymphotropic virus; PN, peripheral neuropathy; OR, odds ratio; vs., versus; EMG, electromyogram.
